# Solubility assessment of single-chain antibody fragment against epithelial cell adhesion molecule extracellular domain in four *Escherichia coli* strains

**DOI:** 10.1186/s43141-021-00126-1

**Published:** 2021-02-04

**Authors:** Fatemeh Sadat Javadian, Majid Basafa, Aidin Behravan, Atieh Hashemi

**Affiliations:** 1 Department of Pharmaceutical Biotechnology, School of Pharmacy, Shahid Beheshti University of Medical Sciences, ValiAsr Avenue, Niayesh Junction, PO Box 14155-6153, Tehran, Iran; 2 Protein Technology Research Center, Shahid Beheshti University of Medical Sciences, ValiAsr Avenue, Niayesh Junction, PO Box 14155-6153, Tehran, Iran

**Keywords:** EpCAM (epithelial cell adhesion molecule), scFv (single-chain variable fragment), Solubility, *E. coli*

## Abstract

**Background:**

Overexpression of the EpCAM (epithelial cell adhesion molecule) in malignancies makes it an attractive target for passive immunotherapy in a wide range of carcinomas. In comparison with full-length antibodies, due to the small size, the scFvs (single-chain variable fragments) are more suitable for recombinant expression in *E. coli (Escherichia coli)*. However, the proteins expressed in large amounts in *E. coli* tend to form inclusion bodies that need to be refolded which may result in poor recovery of bioactive proteins. Various engineered strains were shown to be able to alleviate the insolubility problem. Here, we studied the impact of four *E. coli* strains on the soluble level of anti-EpEX-scFv (anti-EpCAM extracellular domain-scFv) protein.

**Results:**

Although results showed that the amount of soluble anti-EpEX-scFv obtained in BL21^TM^ (DE3) (114.22 ± 3.47 mg/L) was significantly higher to those produced in the same condition in *E. coli* Rosetta^TM^ (DE3) (71.39 ± 0.31 mg/L), and Origami^TM^ T7 (58.99 ± 0.44 mg/L) strains, it was not significantly different from that produced by *E. coli* SHuffle^TM^ T7 (108.87 ± 2.71 mg/L). Furthermore, the highest volumetric productivity of protein reached 318.29 ± 26.38 mg/L in BL21^TM^ (DE3).

**Conclusions:**

Although BL21^TM^ (DE3) can be a suitable strain for high-level production of anti-EpEX-scFv protein, due to higher solubility yield (about 55%), *E. coli* SHuffle^TM^ T7 seems to be better candidate for soluble production of scfv compared to BL21^TM^ (DE3) (solubility yield of about 30%).

## Background

EpCAM (epithelial cell adhesion molecule) is one of the first cancer associated antigens considered as a suitable target for cancer immunotherapy. It is composed of two domains EpEX (an EpCAM extracellular domain-scFv with 265 amino acid residues) and EpICD (an intracellular domain with 26 amino acid residues). Interestingly, Ca^2+^ independent homotypic cell-cell adhesion can be mediated by this molecule during cell proliferation, migration, differentiation, and signaling [1, 2]. Due to EpCAM exclusive overexpression in epithelial-derived neoplasms, it can be considered as a suitable target for many solid tumors and cancer stem cells [3]. Edrecolomab was the first EpCAM-directed monoclonal antibody MAB (monoclonal antibody) approved for human cancers more than 30 years ago. Since then, several other candidates have been assessed in clinical trials [4]. Advances in genetic engineering techniques could facilitate producing recombinant antibody fragments of various sizes and shapes including Fv (variable fragments), Fab (antigen-binding fragments), and scFvs (single-chain variable fragments). ScFv is a non-immunogenic, small arrangement of the functional recombinant antibody fragment which is made up of a heavy V_H_ (chain variable domain) and a V_L_ (light chain variable domain) of an antibody [5, 6]. In contrast to high specificity and affinity, low thermodynamic stability can limit in vivo applications of recombinant scFv fragments. One solution is to transplant the stability of a stable scFv by grafting the CDR (complementarity-determining region) onto a fragment with suboptimal stability. Based on this approach, the binding residues of the anti-EpEX-scFv generated from the parental hybridoma MOC31 were grafted onto the scFv 4D5 framework leading to the formation of a high affinity and very stable 4D5MOC-B scFv [7]. Among different expression hosts, the Gram-negative bacterium *E. coli* (*Escherichia coli*) is preferred as the first choice for laboratory investigations to express recombinant proteins because of its simplicity and fast growth in low cost media as well as the availability of a large number of mutant host strains. However, insolubility is one of the main challenges that need to be overcome for improving the production of biologically active heterologous proteins in this host [8]. To circumvent the solubility problem, several strategies were previously considered including protein secretion to the periplasm of *E. coli*, using weak promoters or solubility-enhancing fusion tags, optimization of the cultivation conditions, and utilization of numerous genetically modified hosts [7]. Because of supplying extra copies of rare tRNAs, reducing endogenous proteases or facilitating disulfide bond formation, various commercially available engineered *E. coli* hosts have been able to promote the expression and solubility of a recombinant protein [9]. For example, the production of soluble TNF-α (tumor necrosis factor α) has been tested in different expression hosts including BL21^TM^ (DE3); BL21^TM^ (DE3)pLysS and Rosetta^TM^. Results showed that soluble TNF-α yield was higher when BL21^TM^ (DE3)pLysS was used as an expression host. However, successful expression and solubility depend on recombinant protein expressed and should be assessed on a case-by-case basis [10], although 4D5MOC-B ScFv fragment was previously expressed in *E. coli* BL21™ (DE3) (2) and *E. coli* Rosetta™ (DE3) (3) strains. In the current study, we evaluated for the first time the effect of four various engineered *E. coli* hosts including *E. coli* BL21^TM^ (DE3), *E. coli* Rosetta^TM^ (DE3), *E. coli* Origami^TM^ (DE3), and SHuffle^TM^ T7 strains on the expression level and solubility of 4D5MOC-B scFv fragment.

## Methods

### Bacterial strains, plasmids, and reagents

The chemically competent *E. coli* (DH5α) (kindly provided by Dr. keramati) was used as host for plasmid preparation and *E. coli* BL21^TM^ (DE3) (kindly provided by Dr. keramati), *E. coli* Rosetta^TM^ (DE3), *E. coli* Origami^TM^ (DE3) (Pasteur institute of IRAN, Tehran, Iran), and *E. coli* SHuffle^TM^ T7 (kindly gifted from Dr. Nematollahi) strains were used as hosts for recombinant scFv expression. All strains were grown on LB (Luria Bertani) medium [1% (w/v) tryptone, and 1% (w/v) NaCl, 0.5% (w/v) yeast extract, pH 7.0] containing antibiotics [ampicillin (100 μg/mL)] when appropriate. All chemicals and reagents used were purchased from standard commercial sources.

### The expression of recombinant anti-EpEX-scFv

The pET22b (+)-anti-EpEX-scFv expression plasmid developed in our previous works was transformed into expression hosts [7]. A single colony of *E. coli* harboring pET22b (+)-anti-EpEX-scFv was inoculated into 3 mL of ampicillin (100 μg/mL)-supplemented LB medium. After overnight constant shaking at 37 °C, the culture was transferred to LB medium supplemented with ampicillin at a ratio of 1∶10. To induce the expression of anti-EpEX-scFv, 0.8 mM IPTG (Isopropyl β-D-1-thiogalactopyranoside) (Sinaclon, Iran) was added to the culture in cell density between 0.7 and 0.9. The mixture was shaken at 24 h at 37 °C**.**

### SDS–PAGE and western blotting

The expression of anti-EpEX-scFv was evaluated via the standard SDS-PAGE (Sodium Dodecyl Sulfate–Polyacrylamide gel electrophoresis) method [7]. After centrifugation at 8000×***g*** for 15 min, the total bacterial pellet was resuspended in 10 mL of the buffer containing 50 mM NaCl, 20 mM Tris HCl pH 7.5, 1 mg/mL lysozyme, 50% glycerol and vortexed. By sonication (400 W for 18 min 20 s ON, 10 s OFF), the lysate was further lysed. The lysate was then centrifuged at 10,000×g for 25 min at 4 °C. Protein samples were separated onto Sodium Dodecyl Sulfate–Polyacrylamide gel electrophoresis (80 V for 5% gel and 150 V for 12% gel). Based on densitometry analysis of polyacrylamide gel bands, the level of the expressed anti-EpEX-scFv was quantified using TotalLab TL120 software (Nonlinear Inc., Durham NC, USA). Western blotting was also carried out for confirmation of the anti-EpEX-scFv expression as a hexahistidine-tagged protein. Extracted proteins separated by 12% SDS-PAGE were then electrophoretically transferred to polyvinylidene fluoride (PVDF) membrane using a wet Transblot (Bio-Rad, USA). The transferred membrane subsequently was blocked in 5% nonfat milk in tris-buffered Saline-Tween (TBST) for 1 h. After incubation with anti-His Tag polyclonal antibody (1/10,000 dilution) (Sigma, UK; Catalog No. H1029) for 1 h, membrane was washed and incubated in HRP (horseradish peroxidase) conjugated anti-mouse immunoglobulin secondary antibody (1/5000 dilution) (Sigma, UK; Catalog No. A0168). The blot was visualized using the ECL (enhanced chemiluminescence) reagent (GE Healthcare, Catalog No. RPN2235). The unstained protein ladder (Thermo Fisher Catalog No. 26610) was used for the protein size assessment **[**11**]**.

### Purification of the soluble portion of recombinant anti-EpEX-scFv

After verification of the presence of anti-EpEX-scFv in supernatant by SDS-PAGE analysis, the supernatant fraction was resuspended in G50 Buffer (20 mM Tris HCl pH 7.5, 50 mM NaCl, 50% glycerol) as a soluble fraction. The resuspended protein was then denatured with buffer (Tris 50 mM, NaCl 50 mM, 1% triton X100, 8 M Urea; pH 8) and subjected to the affinity chromatography column packed with high-capacity Ni–NTA agarose beads under native condition as described by the manufacturer (Qiagen, Netherlands). Using buffers containing 20 mM imidazole, the Ni–NTA column was washed and then the anti-EpEX scFv was eluted from the column by 250 mM imidazole [6].

### Determination of protein concentration

Using the BCA (bicinchoninic acid) assay, the total protein concentrations were quantitatively determined. Based on concentrations of BSA (Bovine Serum Albumin Takara, Japan) standard samples, we first constructed a standard curve which was used as a reference to assess the concentration of the total protein. Total protein was also loaded into a 12% SDS-PAGE gel. Utilizing TL120 software (Nonlinear Inc., Durham NC, USA), the intensity of the gel bands corresponding to anti-EpEX-scFv was analyzed. Depending on estimated intensity, the amount of protein was estimated as a percentage of total protein by TL120. The concentration of recombinant anti-EpEX-scFv can be estimated according to the percentages of anti-EpEX-scFv obtained from TL120 analysis and total protein concentrations obtained from BCA assay [8].

### Statistical analysis

For statistical analysis, GraphPad Prism 6.0 for windows (GraphPad Prism, San Diego, CA, USA) was used. Results are given as mean ± standard deviation (SD) of the mean. Data were analyzed by unpaired, two-tailed Student’s *t* test. *P* < 0.05 was considered statistically significant.

## Results

### The anti-EpEX-scFv protein expression and detection

**Fig. 1:**
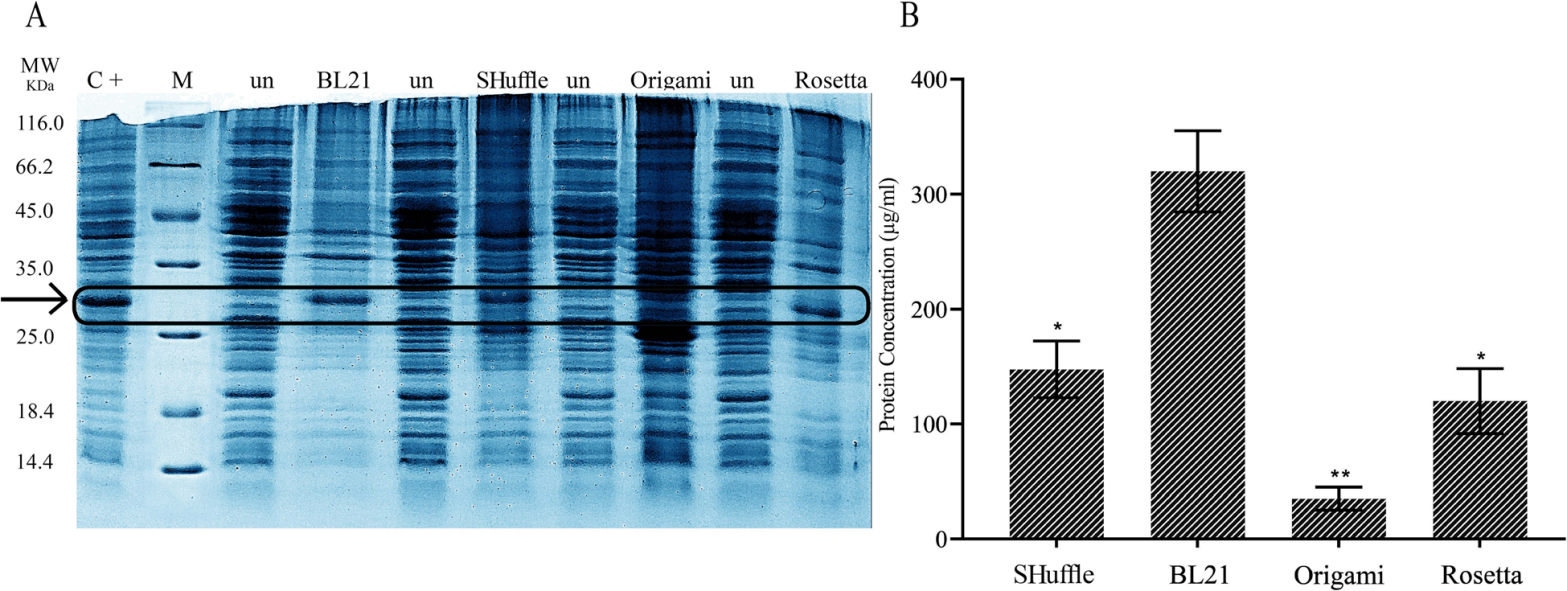
Expression analysis of the antiEpEX-scFv protein using SDS-PAGE. **a** The expression level of the target protein was examined in four *E. coli* strains. The negative control was cells harboring the corresponding plasmid but without induction and the positive control was *E. coli* K-12 BW25113 cells harboring the corresponding plasmid and were inducted by 0.8 mM IPTG at 37 °C for 24 h (C+)(M: protein marker). **b** Protein concentration was quantitatively analyzed using BCA. Results showed that the expression levels were lower in *E. coli* Rosetta^TM^ T7, *E. coli* Origami^TM^ (DE3) and *E. coli* SHuffle^TM^ (DE3) compared with *E. coli* BL21^TM^ (DE3). Data were analyzed by unpaired, two-tailed Student’s *t* test and expressed as the mean ± SD of two experiments (*p < 0.05, **p < 0.01)

**Fig. 2:**
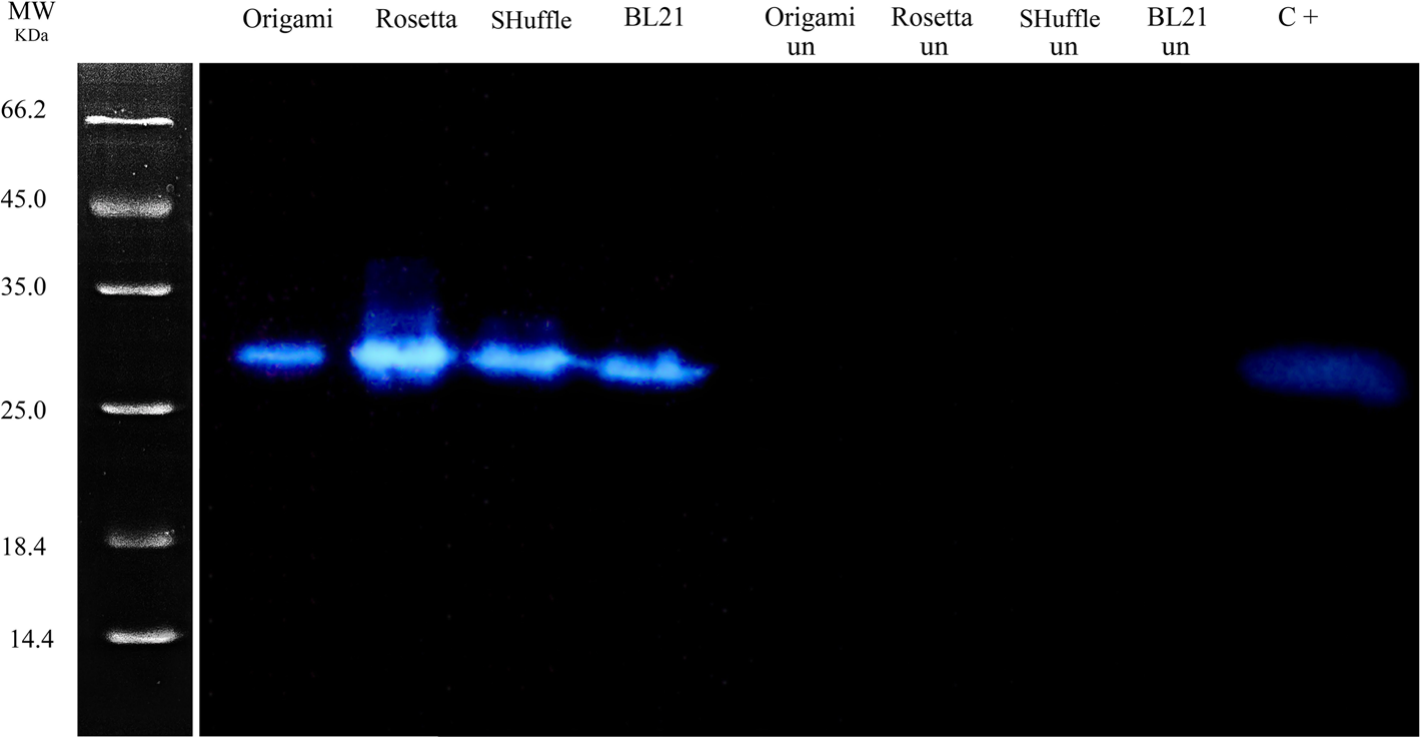
Western blotting analysis of the recombinant antiEpEX-scFv protein. Bacterial lysates of four *E. coli* strains including BL21^TM^ (DE3), SHuffle^TM^ T7, Origami^TM^ (DE3), and Rosetta^TM^ (DE3) before induction (un) and after induction were treated with the anti His tag polyclonal antibody The positive control was *E. coli* K-12 BW25113 cells harboring the corresponding plasmid and were inducted by 0.8 mM IPTG at 37 °C for 24 h (C+)

*E. coli* BL21^TM^ (DE3), *E. coli* Rosetta^TM^ (DE3), *E. coli* Origami^TM^ (DE3), and *E. coli* SHuffle^TM^ T7 competent cells were transformed with the expression pET22b (+) plasmid containing 751 bp anti-EpEX-scFv coding sequence according to the standard heat-shock protocol. Transformants were induced by 0.8 mM IPTG at 37 °C for 24 h. After cell lysis, the anti-EpEX-scFv expression was analyzed via SDS-PAGE. The theoretically expected protein bands with the molecular mass of 30 kDa were detected in all strains compared to the negative controls which are cells harbored the expression plasmid but without the addition of the IPTG (Fig. 1a) (the positive control was *E. coli* K-12 BW25113 cells harboring the corresponding plasmid and were inducted by 0.8 mM IPTG at 37 °C for 24 h (C+) (our previous unpublished data)). The protein expression levels were measured in four strains using the BCA assay. According to BCA results and densitometric analysis of SDS-PAGE bands, a fairly good expression was detected up to 32.8% (318.29 ± 37.3 mg/L) and 17.3% (148.94 ± 29.57 mg/L) of the total protein in *E. coli* BL21^TM^ (DE3) and *E. coli* SHuffle^TM^ (DE3), respectively. Moreover, expression levels were lower in Rosetta^TM^ T7 (123.31 ± 35.59 mg/L) and *E. coli* Origami^TM^ (DE3) (33.25 ± 7.84 mg/L) (Fig. 1b). As shown in Fig. 1b, the highest total protein level was obtained in BL21^TM^ (DE3) (318.29 ± 37.3). The anti-EpEX-scFv expression was more examined by western blotting analysis using a specific anti-his antibody. Results indicated a his-tagged protein with a molecular weight of 30 kDa compared to the negative control in all strains (Fig. 2) (the positive control was *E. coli* K-12 BW25113 cells harboring the corresponding plasmid and were inducted by 0.8 mM IPTG at 37 °C for 24 h (C+) (our previous unpublished data)).

### Influence of different strains on the anti-EpEX-scFv solubility

**Fig. 3:**
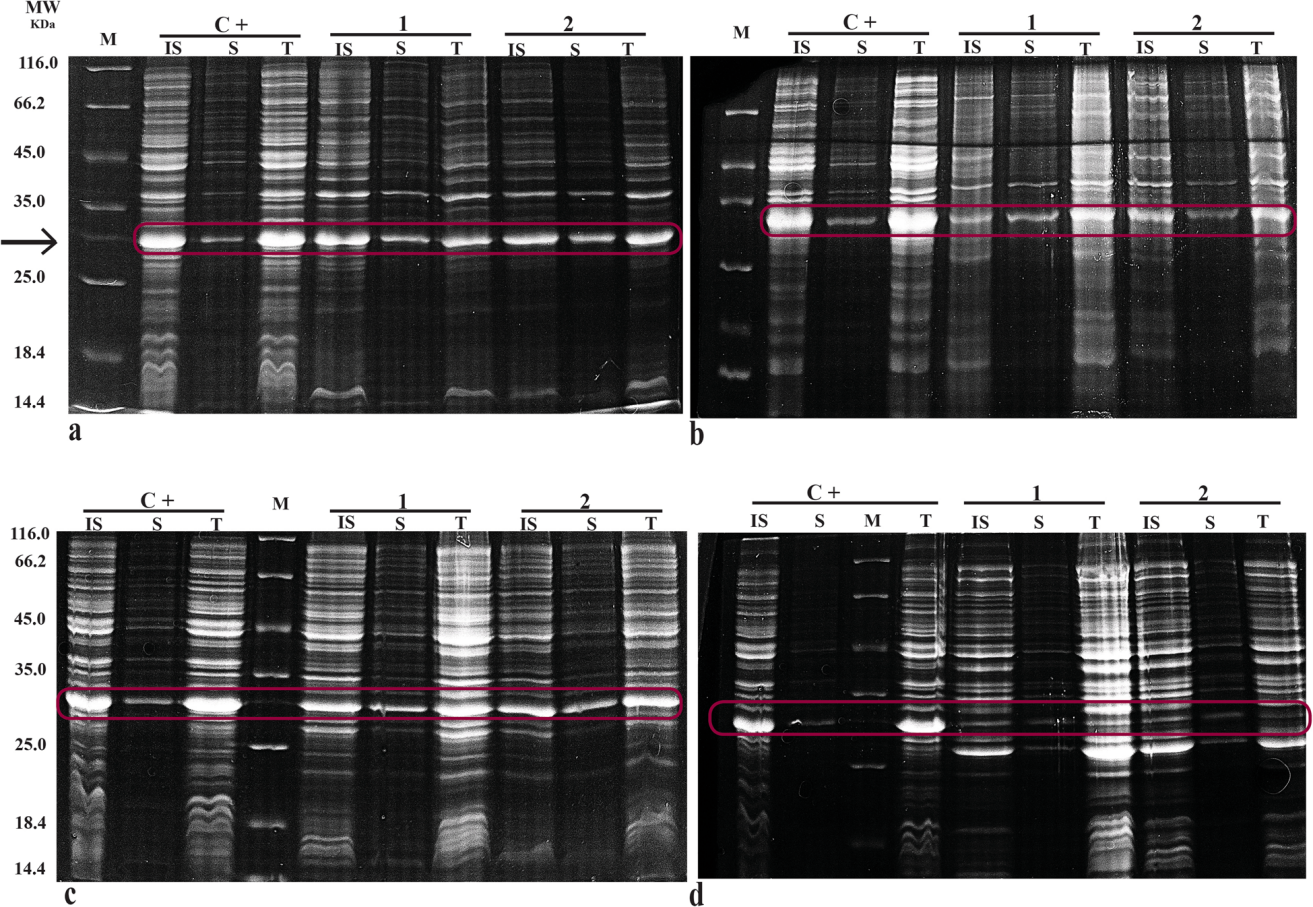
Solubility assessment of anti-EpEX-scFv protein in four hosts. The total (T), soluble (S), and insoluble (IS) fractions of the expressed protein were prepared and subjected to SDS-PAGE. Bands related to anti-EpEX-scFv protein expressed in **a** BL21^TM^ (DE3), **b** Rosetta^TM^, **c** SHuffle^TM^ T7, and **d** Origami^TM^ (DE3) were visualized by coomassie brilliant blue staining. The experiments were performed in duplicates (1) and (2) for each strain. The positive control was *E. coli* K-12 BW25113 cells harboring the corresponding plasmid and was inducted by 0.8 mM IPTG at 37 °C for 24 h (C+)

The data on solubility of anti-EpEX-scFv in four tested strains were compared. Using SDS PAGE, the anti-EpEX-scFv protein distribution was studied in pellet, and supernatant samples. As shown in Fig. 3, the recombinant protein is visually detectable in both soluble and insoluble fractions in all strains including *E. coli* BL21^TM^ (DE3), *E. coli* Rosetta^TM^ (DE3), and *E. coli* SHuffle^TM^ T7, and Origami^TM^ T7 (the positive control was *E. coli* K-12 BW25113 cells harboring the corresponding plasmid and were inducted by 0.8 mM IPTG at 37 °C for 24 h (C+) (our previous unpublished data)).

### Purification of the soluble portion of recombinant anti-EpEX-scFv

**Fig. 4:**
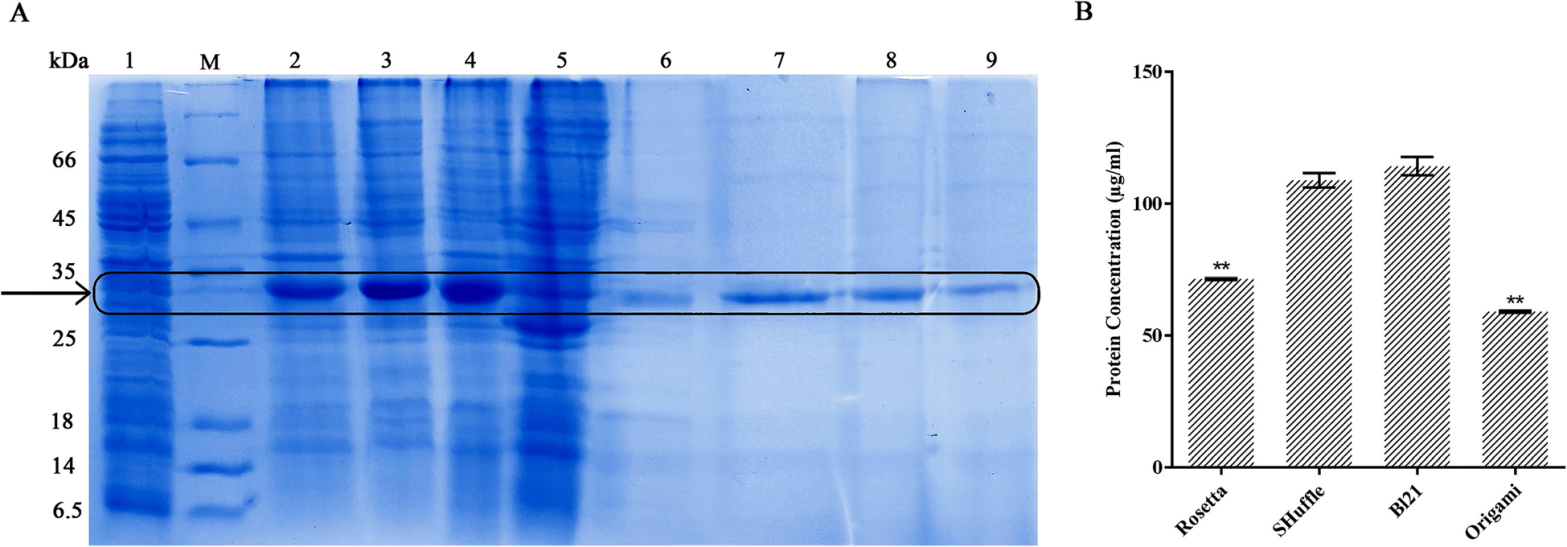
Comparison between the amounts of purified soluble fraction of protein in four *E. coli* hosts. **a** SDS-PAGE analysis of the purified protin, lane 1: negative control harboring empty pET22b, M: molecular weight marker (6.5 to 116 kDa), lanes 2 to 5: induced bacterial lysate in 2: Rosetta^TM^ 3: SHuffle^TM^ T7, 4: BL21^TM^ (DE3), and 5: Origami^TM^ (DE3); lanes 6 to 9: the purified recombinant anti-EpEX-scFv protein from soluble fractions 6: Rosetta^TM^ 7: SHuffle^TM^ T7, 8: BL21^TM^ (DE3), and 9: Origami^TM^ (DE3). **b** Quantitatively protein concentration assessment for soluble fractions after purification. The amount of soluble anti-EpEX-scFv produced by *E. coli* Rosetta^TM^ (DE3) strain and *E. coli* Origami^TM^ T7 was significantly lower than that expressed by BL21^TM^ (DE3). There was no significant difference between the soluble amounts produced by BL21^TM^ (DE3) and SHuffle^TM^ T7. Data were analyzed by unpaired, two-tailed Student’s *t* test and expressed as the mean ± SD of two experiments (**p < 0.01)

Using Ni-NTA affinity chromatography column, the recombinant anti-EpEX-scFv protein was purified from soluble fraction followed by BCA analysis. Although BL21 has the highest volumetric protein production (318.3 mg/L), the amount of soluble anti-EpEX-scFv was obtained in BL21^TM^ (DE3) (114.22 ± 3.47 mg/L) was not significantly different from that produced by *E. coli* SHuffle^TM^ T7 (108.87 ± 2.71 mg/L) (Fig. 4). So, due to higher solubility yield (about 55%), *E. coli* SHuffle can be better candidate for soluble production of scfv compared to BL21^TM^ (DE3) (solubility yield of about 30%). Moreover, the amount of soluble anti-EpEX-scFv produced by the codon bias-adjusted *E. coli* Rosetta^TM^ (DE3) strain (71.39 ± 0.31 mg/L) was significantly lower than that expressed by BL21^TM^ (DE3) and SHuffle^TM^ T7 24 h after induction while was higher than that produced by Origami^TM^ T7 (58.99 ± 0.44 mg/L) strain (Fig. 4). The experiments were performed in duplicates.

## Discussion

Among the available expression hosts, *E. coli* remains the most attractive one for production of heterologous proteins for a variety of downstream utilization. Considerable amounts of soluble protein are required for many analysis techniques such as circular dichroism, crystallography and nuclear magnetic resonance [12]. However, due to high-level expression, many recombinant proteins form inactive insoluble aggregates in the cytoplasm called inclusion bodies which are devoid of biological activities. A high concentration of denaturing agents is needed to solubilize and recover functionally active proteins from these aggregates. This process is a challenging procedure and may result in the loss of secondary structure and poor recovery of bioactive proteins [6, 9]. Moreover, a successful refolding process must be experimentally obtained for each protein. For example, in one study, for optimizing refolding parameters, the effect of more than 200 different buffers on solubilizing of recombinant falcipain-2 IBs was evaluated. They observed that an alkaline buffer containing sucrose or glycerol and glutathione resulted in high yield of solubilized protein [13]. So, developing an efficient approach which can decrease inclusion bodies’ formation without decreasing production yield would reduce the need of additional purification steps and downstream processing costs. Remarkably, to reduce the cost of economic and commercial-scale recombinant protein production, researchers put enormous efforts on elevating expression yield of soluble recombinant proteins [14]. Although a universal approach to circumvent this problem does not exist, various strategies have been described to promote the **in vivo** solubility of the recombinant protein in this bacterial host. Varying several parameters such as fusion partners, post induction temperature, promoters, or *E. coli* strains have been reported to be able to alleviate the insolubility problem and increase the yield of soluble recombinant proteins [15]. To overcome this issue, the effect of engineered strains like *E. coli* Rosetta^TM^ (DE3) (a codon bias-adjusted strain), Origami^TM^ (DE3) (a strain with deletion in thioredoxin and glutathione reductase genes (trxB-, gor-)), SHuffle^TM^ T7 (a strain with deletion in thioredoxin and glutathione reductase genes (trxB-, gor-) and overexpression of cytoplasmic DsbC chaperone), and *E. coli* BL21^TM^ (DE3) (deficient in lon and ompT proteases) on the soluble level of anti-EpEX-scFv was studied here for the first time. Consistently, the expression and solubility of the therapeutic proteins including EPO (erythropoietin), TNFR ED (tumor necrosis factor receptor extra cellular domain), and SK (streptokinase) in four different strains of *E. coli* namely BL21 (DE3), BL21 (DE3) pLys S, BL21 (DE3) Rosetta pLys S, and GJ1158 were compared by Ramkumar et al. [16].

Our results demonstrated that the amount of purified soluble recombinant protein in *E. coli* Rosetta^TM^ (DE3) (71.39 ± 0.31 mg/L) strain is reduced compared to the BL21^TM^ (DE3) (114.22 ± 3.47 mg/L) strain (Fig. 4). This result is in good agreement with a previously published report, where papaneophytou et al. tested different expression hosts including BL21^TM^ (DE3); BL21^TM^ (DE3) pLysS and Rosetta^TM^ to optimize the production of soluble TNF-α in *E. coli*. According to their results, soluble TNF-α yield was comparable in BL21^TM^ (DE3) and BL21^TM^ (DE3) pLysS and was higher compared with Rosetta^TM^ [10]. Consistently, the expression of multiple plant proteins was analyzed in the *E. coli* BL21^TM^ (DE3)-CodonPlus-pRIL as well as the BL21^TM^ (DE3)-pLysS strain (BL), a codon bias-adjusted strain containing augmented levels of tRNAs translating AGA/AGGArg, CUALeu, and AUAIle codons by Rosano et al. Results showed that the expressed plant proteins were mainly insoluble in BL21^TM^ (DE3)-CodonPlus-pRIL strain. On the contrary, in commonly used *E. coli* BL21^TM^ (DE3)-pLysS strain, they had higher solubility [17]. Moreover, in Fahnert et al. study, although overexpression of argU tRNA during α-glucosidase expression in *E. coli* could increase translation rate, but aggregation was also stimulated [18]. Based on these reports along with our results, the expressed recombinant proteins can become more insoluble in codon bias-adjusted *E. coli* strains compared to the parent strain. This phenomenon was explained by Zhang et al. They reported that slow-translation could cause a protein chain to be discontinuously elongated which might affect the protein folding. They showed that protein folding events could be facilitated by modulation of the translational speed. At specific sites, rare codons are thought to slow the RNA translation and let the protein to be folded sequentially which may result in an increase in the number of protein molecules with proper fold. Consequently, utilization of commercial bacterial strains with augmented tRNA level can increase the translation speed and consequently, protein aggregation [19].

In this study, the soluble anti-EpEX-scFv expressed in *E. coli* Origami^TM^ (58.99 ± 0.44 mg/L) was significantly lower than that in BL21^TM^ (DE3) (Fig. 4). This result is in good agreement with a previously published report, where the impact of three *E. coli* expression strains and twelve fusion tags on the solubility of 28 DRPs (disulfide-rich proteins) of variable size (from 25 to 122 aa) has been studied in a high throughput screening approach. The screening showed that when the strain BL21^TM^ (DE3) pLysS was used as a host, soluble expression was identified in 196 conditions among 336 conditions tested per strain whereas only 44 conditions led to soluble expression in Origami B (DE3) pLysS strain (15). Consequently, BL21^TM^ (DE3) seems to be more suitable than the Origami^TM^ strain for the soluble production of anti-EpEX-scFv or DRPs. Altered growth parameters might be the reason for lower soluble expression level in Origami^TM^ (DE3) strains compared to other *E. coli* strains like BL21^TM^ (DE3) [15].

Based on results obtained in the current study, despite the higher yield of protein expression in BL21 (DE3), SHuffle^TM^ T7 exhibited higher yield of protein solubility (about 55%). Our results are consistent with the Safarpour et al. study in which the expression of tumor necrosis factor alpha (TNF-α) was compared in BL21 (DE3) and SHuffle^TM^ T7. In their study, TNF-α expressed in SHuffle^TM^ T7 showed 1.5-fold higher disulfide bind formation compared with that expressed in BL21^TM^ (DE3) whereas BL21^TM^ (DE3) exhibited a higher yield of TNF-α expression than SHuffle^TM^ T7 [20]. Furthermore, a recent study in which the soluble expression level of GM-CSF (Granulocyte-macrophage colony-stimulating factor) was compared in three *E. coli* strains including Origami™ 2, (DE3), BL21 (DE3), and SHuffle^TM^ T7 showed higher yield of soluble GM-CSF in SHuffle^TM^ T7 in comparison with BL21^TM^ (DE3) [9]. Interestingly, the highest soluble expression of recombinant human fibroblast growth factor 1 was also obtained in SHuffle^TM^ T7 in Nasiri et al. study in which the expression of recombinant protein was compared in three strains. As it is anticipated chaperone properties of DsbC as well as oxidative environment can provide the correct folding of expressed proteins in the cytoplasm of SHuffle^TM^ T7 [21].

## Conclusions

Solubilization of inclusion bodies into bioactive proteins is a challenging and difficult task which contribute to increase in production cost and decrease in production yield. Therefore, for economic and commercial-scale recombinant protein production, we need to develop methods for soluble expression of recombinant proteins [22].

In the current study, investigating the effect of engineered *E. coli* strains on the soluble level of anti-EpEX-scFv showed that the amount of soluble protein obtained in BL21^TM^ (DE3) (114.22 ± 3.47 mg /L) was significantly higher to those produced in the same condition in *E. coli* Rosetta^TM^ (DE3) (71.39 ± 0.31 mg/L), and Origami^TM^ T7 (58.99 ± 0.44 mg/L) strains while was not significantly different from that produced by *E. coli* SHuffle^TM^ T7 (108.87 ± 2.71 mg/L). So, due to higher solubility yield (about 55%), *E. coli* SHuffle^TM^ T7 seems to be better candidate for soluble production of scfv compared to BL21^TM^ (DE3) (solubility yield of about 30%).

## Data Availability

All data generated or analyzed during this study are included in this published article

## Abbreviations

EpCAM: Epithelial cell adhesion molecule
ScFvs: Single-chain variable fragments
*E. coli*: *Escherichia coli*
Anti-EpEX-scFv: Anti-EpCAM extracellular domain-scFv
MAB: Monoclonal antibody
VH: Chain variable domain
VL: Light chain variable domain
CDR: Complementarity-determining region
LB: Luria Bertani
IPTG: Isopropyl β-D-1-thiogalactopyranoside
SDS-PAGE: Sodium Dodecyl Sulfate–Polyacrylamide gel electrophoresis
ECL: Enhanced chemiluminescence
BCA: Bicinchoninic acid
BSA: Bovine serum albumin
DRPs: Disulfide-rich proteins
